# Suicide mortality data from the Italian police during the COVID-19 pandemic

**DOI:** 10.1186/s12991-021-00350-6

**Published:** 2021-05-07

**Authors:** Sergio Garbarino, Michele Fornaro, Rita Messina, Maurizio Pompili, Fabrizio Ciprani

**Affiliations:** 1 State Police Health Service Department, Ministry of the Interior, 00198 Rome, Italy; 2 Department of Neuroscience, Rehabilitation, Ophthalmology, Genetics and Maternal/Child Sciences (DINOGMI), University of Genoa, Genoa, Italy; 3 Section of Psychiatry, Department of Neuroscience, University School of Medicine “Federico II,”, Naples, Italy; 4 Department of Neurosciences, Mental Health and Sensory Organs, Suicide Prevention Center, Sant’Andrea Hospital, Sapienza University, 00185 Rome, Italy

**Keywords:** Suicide, Police officers, COVID-19, Prevention

## Abstract

Suicide is a major public health issue worldwide, with about 880,000 dying annually for such a cause. The COVID-19 pandemic has led to severe social disruption both from health and economic standpoints. Law enforcement personnel have been significantly involved in helping to face the many difficulties due to the pandemic. Police officers have been subjected to further stress from managing social restrictions imposed by governments to contain the pandemic. The Italian State Police steadily approximate 100,000 people aged 25–65 years: 14 subjects (mean age 43.64 years) died by suicide in 2015, 7 (mean age 47.5 years) in 2016, 13 (mean age 45.62 years) in 2017, 10 (mean age 48.1 years) in 2018, 18 (mean age 46.78 years) in 2019, and 12 (mean age 52 years) in 2020. Our records excluded significant changes in suicide incidence rate within 2015–2020 (till December 2020). However, the COVID-19 pandemic spread faster in Italy than in other regions, meaning that the Italian State Police possibly faced prolonged, intense stress. Suicide prevention, therefore, remains a priority, especially during this difficult time.

## Background

Suicide is a major public health issue worldwide, with about 880,000 dying annually for such a cause. It is a multifactorial phenomenon with various variables contributing to the precipitation of one individual wishing to die. The COVID-19 pandemic has led to severe social disruption from health and economic standpoints and increased prevalence of depression symptoms, assessed using the Patient Health Questionnaire–9, in the US adults more than threefold 8.5% before COVID-19 to 27.8% during COVID-19 [1]. Several reports indicated the need to be vigilant during the worldwide health emergency for a possible increase in mental health problems [2] and suicide deaths [3, 4]. Law enforcement personnel have been significantly involved in helping to face the many difficulties due to the pandemic. There is, therefore, the need to explore if suicide rates among police officers changed as a result of the pandemic.

At this time, results regarding suicide rates in the general population are still mixed [5]. According to a recent report by Leske et al. [6], no overall change in Queensland’s suspected suicide rate since the declaration of a local public health emergency (PHED) occurred from January 29 up until late August 2020. This study is prime, since it is the first to consider pre-COVID trends in suicide mortality. Sakamoto et al. [7] explored suicide rates in Japan during 2020. They found that compared with previous years, suicide rates in Japan in 2020 increased in October and November for men and in July through November for women.

Police officers have been subjected to further stress from managing social restrictions imposed by governments to contain the pandemic. While information about the mortality rates due to suicide among people actively serving in a national police corps is rarely released to the public, we can disclose the rates of suicide among the Italian State Police during 2015–2020.

The Italian State Police steadily approximate 100,000 people aged 25–65 years: 14 individuals (males = 13, females = 1; mean age = 43.64 years) committed suicide in the year 2015. Correspondingly, 7 people (males = 7, females = 1; mean age = 47.5 years) in 2016; 13 people (males = 13, mean age = 45.62 years) in 2017; 10 people (males = 10, mean age = 48.1 years) in 2018; 18 people (males = 16, females = 2, mean age 46.78 years) in 2019, and 12 people (males = 12, mean age = 52) in 2020 committed suicide.

While official data for 2020 for the general Italian population are still unavailable, we could not adjust for potential confounders; we feel that the present information is worth reporting. Considering the small size of our sample, our records would excluded significant changes in suicide incidence rate within 2015–2020 (till December 2020). However, the COVID-19 pandemic spread faster in Italy than in other regions, meaning that the Italian State Police possibly faced prolonged, intense stress.

Although data indicating no significant increase in suicides in Queensland during 2020 compared to 2015–2019 annual records and suicide deaths declined by 5.6% from 2019 to 2020 in the US [8], confirmation is mandatory for the complex interactions between self-harm [9], suicide due to stressful events [10], mental health [11], and COVID-19 pandemic, especially considering the compelling need for high-quality studies already prompted-out by Stanley et al. [12].

From the beginning of the pandemic, a task force of international experts on suicide prevention started exploring the issue and provided some guidelines for confronting suicide risk during COVID-19 pandemic [3, 13]. It became clear that the pandemic's impact on suicide may have different pathways according to the different geographic areas and socio-cultural patterns. Overall, this problematic time produced a great deal of human misery [1]. During the 2008 economic crisis, there was a 12% increase in suicide deaths among Italian males aged 25–69, namely those in the labour market [14]. The COVID-19 pandemic closed down most commercial activities, and entire sectors of the economy now struggle to survive.

## Conclusion

The worldwide COVID-19 pandemic and efforts to contain it represent a further threat, and we must recognize the pandemic of mental and behavioral illness that will quickly follow it, and implement the steps needed to mitigate it [2]. Suicide prevention remains a priority, especially during this difficult time [4]. Despite everything, it can be a source of opportunities if viewed in light of the efforts made to implement preventive measures [3, 15]. Such efforts should also be extended to different sub-populations that may have specific unmet needs, such as youths, elderly, or first-line responders that may impact life-threatening facing situations.

## Data Availability

Not applicable.
